# Predictors of Cervical Cancer Screening Adherence in the United States: A Systematic Review

**Published:** 2014-01-01

**Authors:** Karen Limmer, Geri LoBiondo-Wood, Joyce Dains

**Affiliations:** From The University of Texas MD Anderson Cancer Center, Houston, Texas

## Abstract

Cervical cancer incidence rates have decreased dramatically since the implementation of the Papanicolaou (Pap) smear. Nevertheless, the American Cancer Society (ACS) estimates for 2013 predicted more than 12,000 new cases of cervical cancer in the United States. Given that some subpopulations in the United States are at a higher risk for cervical cancer than others, efforts to increase screening adherence are warranted. Many studies have explored the demographics of underscreened women, but no systematic reviews of screening demographics in adult US women were identified in the past 10 years, after release of the 2002 ACS cervical cancer screening guidelines. Knowledge of adherence to these guidelines becomes important as new guidelines were developed and released in 2012. The purpose of this systematic review of relevant studies was to identify factors that predict the use of cervical cancer screening in US women. Variables found to be significantly associated with adherence to screening included education, financial status, acculturation, psychosocial issues, and marital status. Using this information, nurse practitioners and other providers can target specific at-risk populations to increase screening by educating women about the need for cervical cancer screening and ensuring access to methods for prevention and early detection of the disease.

The American Cancer Society (ACS) estimates that more than 12,000 new cases of cervical cancer were diagnosed in 2011 and that more than 4,000 patients with cervical cancer died from their disease (American Cancer Society, 2013). Nonetheless, cervical cancer incidence rates have decreased dramatically since the implementation of the Papanicolaou (Pap) cervical cancer screening test, or smear, in the 1940s (Elliott, 2007). The Pap smear facilitates such early detection of precancerous cervical lesions and established cervical cancer that with subsequent intervention, this cancer can often be prevented or even successfully treated (National Cancer Institute [NCI], 2011a). Given the clear treatment and prevention benefits conferred by Pap smear screening, further efforts to understand the barriers to screening and to increase screening participation among women are clearly warranted.

Moreover, although all women are at risk for cervical cancer, some subpopulations in the United States remain at higher risk than others, and particular attention should be paid to these at-risk populations and attempts made to increase their adherence to current cervical cancer screening guidelines. The incidence of cervical cancer is higher in Hispanic women (12.2 per 100,000) than in Caucasian women (8.0 per 100,000), and the rate of death from cervical cancer is two times higher in African American women than in Caucasian women (National Cancer Institute, 2011b; 2013). Women who smoke, those who are infected with human immunodeficiency virus (HIV), and those who have been exposed to diethylstilbestrol (DES) also have a higher risk of developing and dying of cervical cancer (NCI, 2011c, 2011d).

## Background and Current Screening Guidelines

In the 1920s, George N. Papanicolaou suggested that a sample of cervical cells could be used to detect cervical cancer. In 1941, he published a groundbreaking paper on the cervical smear, which led to controversy, more research, and ultimately, a 1945 recommendation for the use of Pap smears by the ACS (Papanicolaou & Traut, 1997). Cervical cancer death rates decreased substantially with the integration of the Pap smear into routine care, and this test is now universally accepted as the gold standard for detecting precancerous cervical lesions and early cervical cancer. Between 2002 and 2005, 86% of adult women in the United States received at least one Pap smear (Elliott, 2007).

Guidelines for initiation and cessation of cervical cancer screening via Pap smear have varied by recommending authority over the years. The most recent ACS cervical cancer screening guidelines were released in March 2012 (ACS, 2012a). The ACS, as well as the U.S. Preventive Services Task Force (USPSTF), per new 2012 guidelines, and the American Congress of Obstetricians and Gynecologists (ACOG), per new 2013 guidelines, all recommend that women should begin participating in cervical cancer screening at and not before 21 years of age and that subsequent screening should occur every 3 years until age 29.

The ACS, USPSTF, and ACOG all now recommend that the screening interval may be extended to every 5 years for women aged 30 to 65 if human papillomavirus (HPV) testing is used in conjunction with Pap smears. Some types of HPV infection can increase a woman’s risk of developing cervical cancer (ACS, 2012b). None of the three agencies recommends routine screening for HPV in women younger than age 30.

The ACS (2012a), USPSTF (2012), and ACOG (2013) recommend ending screening at age 65 and discontinuing screening in women who have had a hysterectomy to treat benign conditions. These screening guidelines are recommendations for women with normal results only. All three recommending bodies have more specific guidelines for women who have had abnormal Pap smears (ACOG, 2013; ACS, 2012a; USPSTF, 2012).

The detailed guidelines indicate that almost all adult women should be participating in screening and that some women should be screened more frequently than others. However, some women are either not participating in screening or are not adhering to the guidelines based on their age or risk category. Exact adherence is difficult to determine, as recommendations for the timing of Pap smears has historically varied among recommending authorities. A woman may be considered adherent if she follows a cervical cancer screening schedule, based on ACS, USPSTF, and/or ACOG guidelines, with reasonable diligence.

Many studies have addressed which women are not being screened for cervical cancer, demographic characteristics, screening participation, and barriers to screening. However, to our knowledge, no systematic reviews of cervical cancer screening demographics in US adult women have been conducted in the past 10 years, since the release of the 2002 ACS cervical cancer screening guidelines. Knowledge of adherence to this set of guidelines becomes important, as new ACS guidelines were developed and released in March 2012. With better understanding of predictors of adherence to the 2002 guidelines, advanced practitioners may facilitate improved observance of the new guidelines.

Thus, we systematically reviewed the literature since 2002 on predictors of use of cervical cancer screening among US women. Although only studies that were published since 2002 were included, some studies that collected data before 2002 were also included, as eliminating them would have excluded all studies on African American women, which is a high-risk group, as well several studies with clinical implications.

## Methods

The following electronic databases were searched: the Cochrane Collection, Ovid, PubMed, Embase, and the Cumulative Index to Nursing and Allied Health Literature (CINAHL). The following search terms were applied: vaginal smear*, Pap* smear*, cervical cancer screen*, patient compliance, and patient adherence. The asterisks represent root words. The databases include these root words with all possible suffixes in search results. The search results were limited to articles that were published between 2003 and 2011, written in English, and pertained to studies of adult humans. The publication date range was based on the years that the cervical cancer screening guidelines released by the ACS in 2002 were in effect.

Multiple inclusion and exclusion criteria were applied. Studies must have been performed in the United States, as health-care systems and screening guidelines differ from country to country, thereby making comparisons difficult. Studies that focused mainly on cervical cancer screening were included; however, those with only a broad focus on cervical cancer screening in addition to breast and/or colorectal cancer screening and/or other health initiatives were excluded. Studies of associations among characteristics, demographics, or beliefs of women who were or were not participating in cervical cancer screening were included; however, studies that reported the effects of interventions on participation rates were excluded, as interventions were not the focus of this review.

Studies that included only adolescents or only older adults were excluded, as the 2002 guidelines were not well defined in these age groups. Studies with subjects who had already been diagnosed with cervical cancer or who had abnormal cervical cytology were excluded, as were studies of follow-up of abnormal Pap smears, as the screening criteria for these populations are unique. Studies related to practitioner compliance with screening of patients, as opposed to patient adherence with guidelines, were excluded. Studies that only included women who were already following screening guidelines were also excluded, as were studies that assessed cervical cancer screening rates to predict adherence to other types of screening. The literature search yielded 569 articles, 545 of which were eliminated on the basis of inclusion and exclusion criteria; thus, 24 articles were included in the review and were grouped by population assessed.

## Results

Common findings within population groups as well as study strengths and weaknesses are discussed first, followed by a discussion of common findings across population groups. A summary of post-2002 published studies of factors associated with cervical cancer screening adherence by population group is presented in the Table.1

**Table 1:**
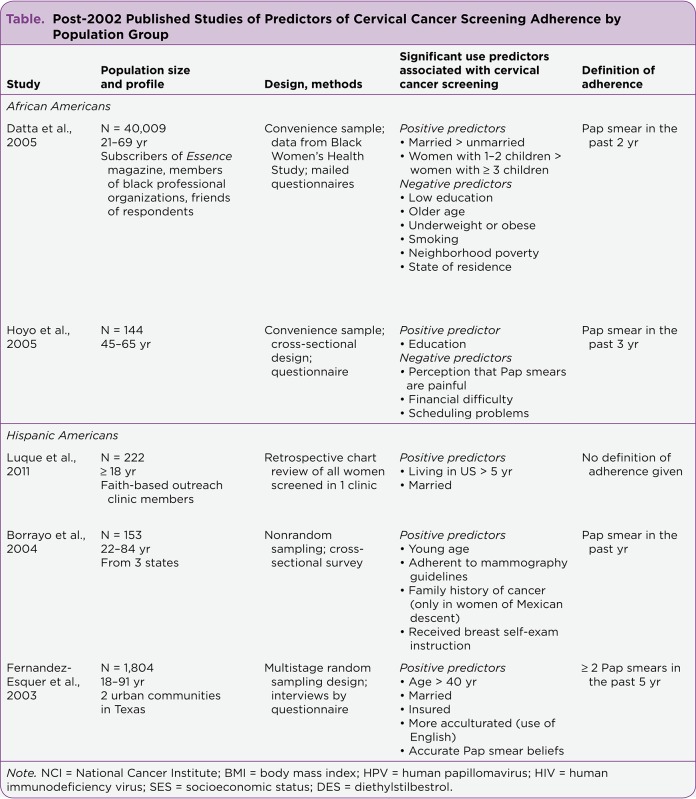
Table 1. Post-2002 Published Studies of Predictors of Cervical Cancer Screening Adherence by Population Group

**Table 2:**
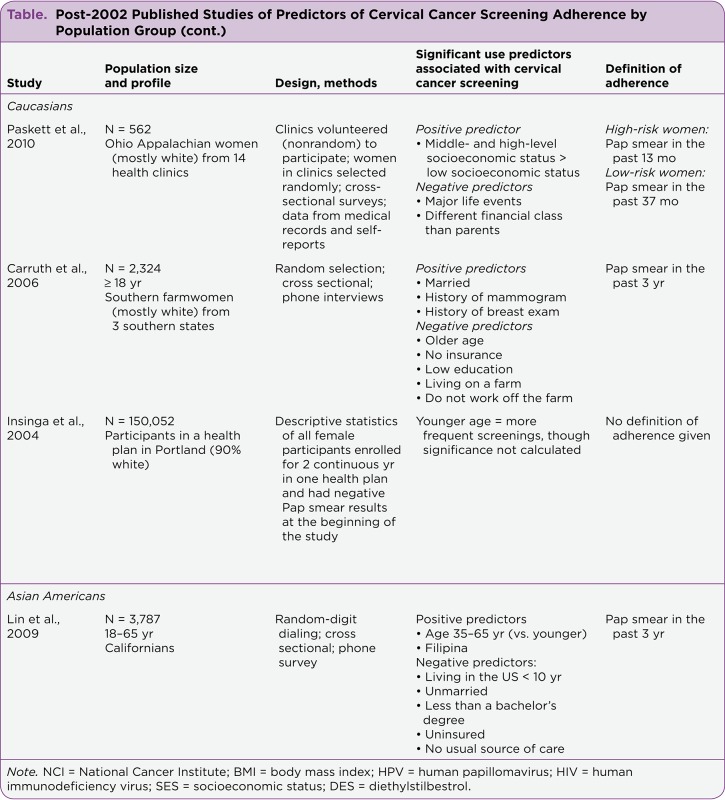
Table 2. Post-2002 Published Studies of Predictors of Cervical Cancer Screening Adherence by Population Group

**Table 3:**
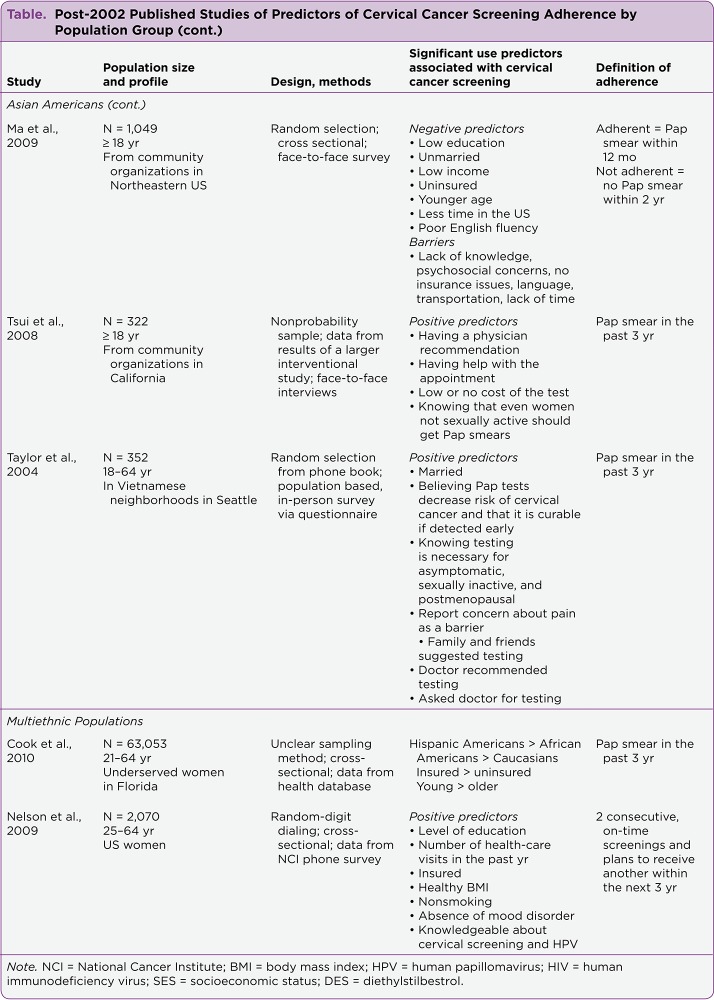
Table 3. Post-2002 Published Studies of Predictors of Cervical Cancer Screening Adherence by Population Group

**Table 4:**
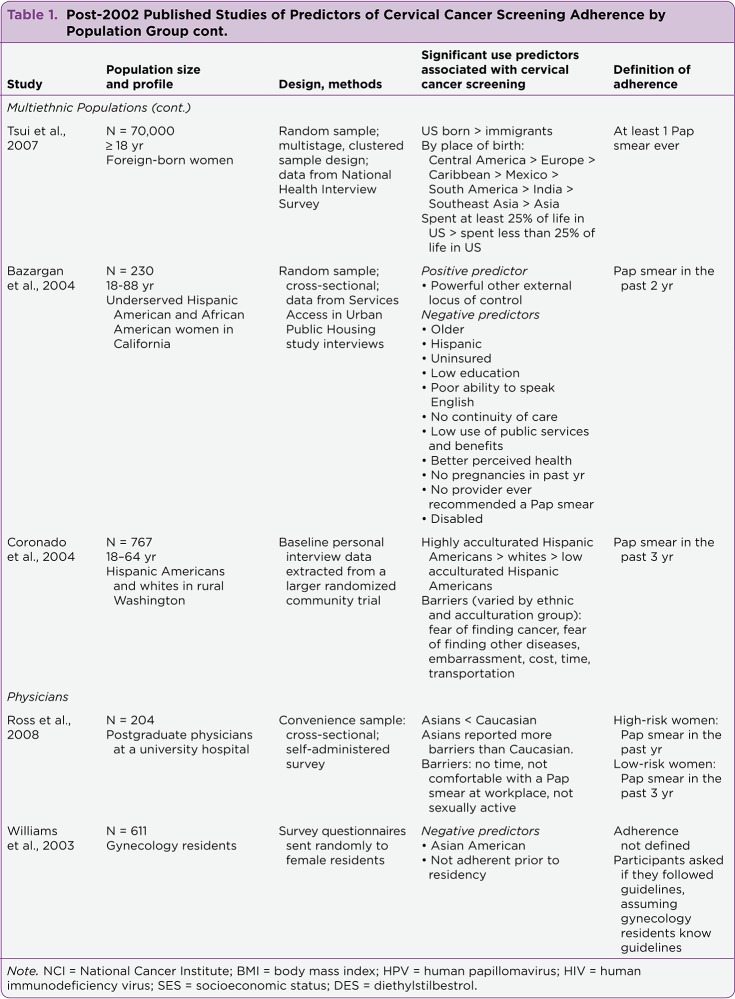
Table 4. Post-2002 Published Studies of Predictors of Cervical Cancer Screening Adherence by Population Group

**Table 5:**
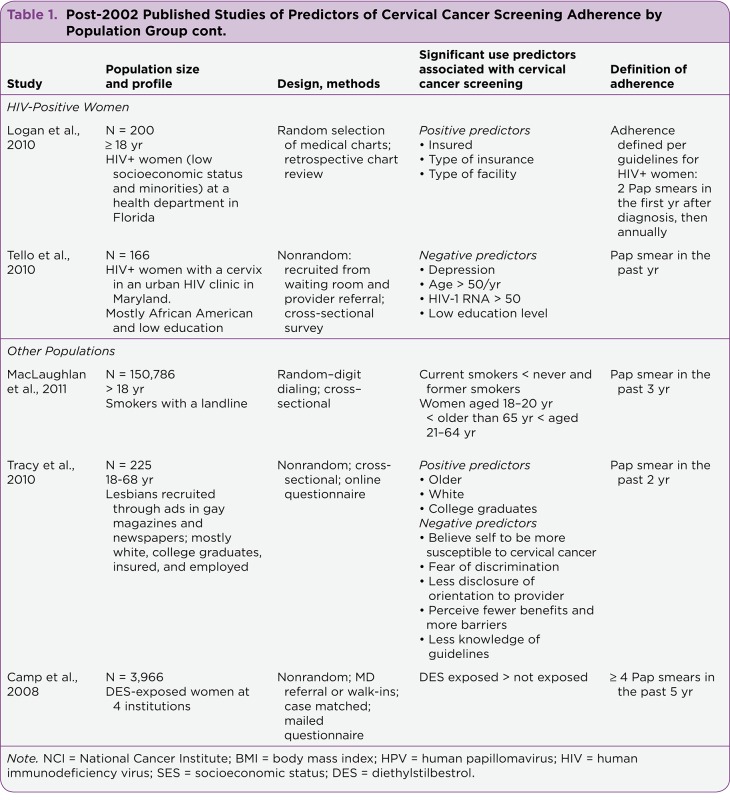
Table 5. Post-2002 Published Studies of Predictors of Cervical Cancer Screening Adherence by Population Group

**African Americans** 

Of the two studies that assessed screening behavior in African American women, both indicated that higher education level was significantly and strongly positively associated with screening adherence (Datta et al., 2005; Hoyo et al., 2005). Both also acknowledged that financial difficulty was a negative predictor of screening adherence. In their study, Datta et al. (2005) included a large sample of 40,009 respondents identified from the Black Women’s Health Study, whereas the study by Hoyo et al. (2005) included only 144 subjects. According to Datta et al. (2005), their study included a sample of women who were more educated than most African American women.

Although both studies relied on convenience sampling and both used data acquired before the release of the 2002 ACS cervical cancer screening guidelines, these important variables seem key to adherence. Datta et al. (2005) and Hoyo et al. (2005) differed in their definitions of adherence to cervical cancer screening. Datta et al. (2005) defined adherence as having received a Pap smear within the 2 years prior to data collection, whereas Hoyo et al. (2005) defined adherence as having received a Pap smear within the 3 years prior to data collection.

**Hispanic Americans** 

Two variables that were positively associated with screening adherence emerged in two of the three studies of Hispanic American women: marriage (Fernandez-Esquer, Espinoza, Ramirez, & McAlister, 2003; Luque et al., 2011) and knowledge of self-care, meaning that women received instructions for conducting breast self-examinations (Borrayo, Thomas, & Lawsin, 2004) and had accurate Pap smear beliefs, as measured by a 12-item questionnaire (Fernandez-Esquer et al., 2003).

Borrayo et al. (2004) and Fernandez-Esquer et al. (2003) reported different outcomes regarding the effects of age on screening adherence. Borrayo et al. (2004) found that "women who were, on average, younger were more likely to have obtained a recent Pap smear, while women who were, on average, older were less compliant" (p. 22). Fernandez-Esquer et al. (2003) found that age > 40 was positively associated with screening adherence.

The study by Fernandez-Esquer et al. (2003) had the largest sample (1,804 participants); the studies by Luque et al. (2011) and Borrayo et al. (2004) included 222 and 153 participants, respectively. Fernandez-Esquer et al. used a multistage random sampling design, whereas Borrayo et al. did not use random sampling, and Luque et al. included all women receiving care at the participating clinic.

One limitation of the study by Fernandez-Esquer et al. is that it included data from the 1990s. Of note, Luque et al. did not define adherence and did not clearly describe how correlations were calculated. Borrayo et al. defined adherence as having received a Pap smear within the year preceding the study, whereas Fernandez-Esquer et al. defined adherence as having received at least 2 Pap smears within the 5 years prior to data collection.

**Caucasians** 

In studies of primarily Caucasian populations, financial and insurance statuses were found to be positively associated with adherence to screening, and age was negatively correlated with adherence (Carruth, Browning, Reed, Skarke, & Sealey, 2006; Insinga, Glass, & Rush, 2004; Paskett et al., 2010). The studies by Insinga et al. (2004) and Carruth et al. (2006) included large samples of 150,052 and 2,324 participants, respectively, whereas the study by Paskett et al. (2010) included a comparatively smaller sample of 562 participants. Carruth et al. (2006) and Paskett et al. (2010) used random selection of participants, and Insinga et al. (2004) included all eligible women (with negative Pap smear results at the beginning of study) who had been enrolled in the Portland health plan for 2 continuous years.

Of note, the results reported by Insinga et al. (2004) were based on percentages and did not include any inferential analysis. Also, Insinga et al. (2004) collected data from 1997 to 2002, and Carruth et al. (2006) did not specify when data were collected, so it is unclear whether data were collected before 2002.

Paskett et al. (2010) defined adherence to screening guidelines according to risk category, which was based on multiple health behaviors. Women were classified as adherent if they had undergone Pap smear testing in the 13 months prior to data collection for high-risk women or the 37 months prior to data collection for low-risk women. Carruth et al. (2006) defined adherence as having undergone cervical cancer screening within the 3 years prior to data collection, whereas Insinga et al. (2004) did not report a definition of adherence.

**Asian Americans** 

All four studies of Asian Americans showed that education, or correct knowledge of facts related to screening, was positively associated with participation in screening (Lin et al., 2009; Ma et al., 2009; Taylor et al., 2004; Tsui & Tanjasiri, 2008). Three of these studies revealed an association between adherence and a financial variable, including insurance status, income, and cost of the test (Lin et al., 2009; Ma et al., 2009; Tsui & Tanjasiri, 2008). Three studies found that marriage was positively associated with screening adherence (Lin et al., 2009; Ma et al., 2009; Taylor et al., 2004), and two studies found that older age was positively associated with screening adherence (Lin et al., 2009; Ma et al., 2009). Two studies found that English fluency and duration of US residency were also associated with screening adherence (Lin et al., 2009; Ma et al., 2009).

The studies by Lin et al. (2009) and Ma et al. (2009) included relatively large samples of 3,787 and 1,049 participants, respectively, whereas the studies by Tsui and Tanjasiri (2008) and Taylor et al. (2004) included smaller samples of 322 and 352 participants, respectively. Lin et al., Ma et al., and Taylor et al. used random sampling methods, whereas Tsui and Tanjasiri used nonprobability sampling. Lin et al., Tsui and Tanjasiri, and Taylor et al. defined screening adherence as having received a Pap smear in the 3 years prior to study enrollment, whereas Ma et al. defined adherence as having received a Pap smear within the 12 months prior to study enrollment. Of note, Lin et al. and Taylor et al. collected data before 2002.

**Multiethnic Populations** 

Being insured was positively correlated with screening participation in three of the five studies of multiethnic populations (Bazargan, Bazargan, Farooq, & Baker, 2004; Cook et al., 2010; Nelson, Moser, Gaffey, & Waldron, 2009), but cost was found to be a barrier to screening participation in only one study (Coronado, Thompson, Koepsell, Schwartz, & McLerran, 2004). Acculturation measured in multiple ways—including being born in the United States, longer duration of US residency, and English fluency—was correlated with screening adherence in three of the five studies (Bazargan et al., 2004; Coronado et al., 2004; Tsui, Saraiya, Thompson, Dey, & Richardson, 2007). Results in two of the studies indicated that younger women were more likely to undergo screening (Bazargan, et al., 2004; Cook et al., 2010). Education also emerged as key in two of the studies (Bazargan et al., 2004; Nelson et al., 2009).

Cook et al. (2010) and Tsui et al. (2007) found that ethnicity was associated with participation in screening. Cook et al. found that among women living in the United States, Hispanic American women were more likely to undergo screening than African American and Caucasian women. Tsui et al. found that among women living in the United States, immigrants from Central America were more likely to undergo screening than were their foreign-born counterparts from other areas, including Europe, the Caribbeans, Mexico, South America, India, and Asia. In contrast with Cook et al., Bazargan et al. (2004) found that Hispanic Americans were less likely to be screened than African Americans were. Nelson et al. (2009) and Bazargan et al. (2004) found that an element of continuity of care was associated with adherence to screening. The former reported that the number of health-care visits in the past year was associated with screening adherence, and the latter reported that continuity of care and provider recommendations were associated with screening adherence.

Another common finding among these studies was that psychosocial issues may play a role in screening adherence. Nelson et al. (2009) found that the absence of a mood disorder was a positive predictor of adherence. Bazargan et al. (2004) found that a powerful others external locus of control, according to the Multidimensional Health Locus of Control scales (Wallston, Wallston, & DeVellis, 1978), was a predictor of adherence. Coronado et al. (2004) found that fear of finding cancer or other diseases was a barrier.

The largest studies in this group, those by Cook et al. (2010) and Tsui et al. (2007), included samples of 63,053 and 70,000 participants, respectively. The other studies had samples of 2,070 participants (Nelson et al., 2009), 230 participants (Bazargan et al., 2004), and 767 participants (Coronado et al., 2004). Random sampling was used in all of the studies except the Cook et al. study, in which the sample selection was not clearly described.

The definition of adherence varied among the studies. Bazargan et al. (2004) defined adherence as having received a Pap smear within the 2 years prior to data collection; Cook et al. (2010) and Coronado et al. (2004) defined adherence as having received a Pap smear within the 3 years prior to data collection. Tsui et al. (2007) defined adherence as ever having a Pap smear, and Nelson et al. (2009) defined adherence as having 2 consecutive, on-time screenings and plans to receive another within the 3 years after the study was conducted. Bazargan et al. (2004), Coronado et al. (2004), and Tsui et al. (2007) used data collected before 2002.

**Physicians** 

Two studies sought to determine rates of screening adherence in patients who are physicians. Both found that Asian American physicians were less likely to undergo recommended cervical cancer screening than were physicians of other ethnicities, specifically Caucasians (Ross, Nunez-Smith, Forsyth, & Rosenbaum, 2008; Williams, Santoso, Ling, & Przepiorka, 2003). The study by Williams et al. (2003) included 611 participants, compared with 204 participants in the study by Ross et al. (2008). Williams et al. (2003) used random sampling, whereas Ross et al. (2008) used convenience sampling. Williams et al. (2003) did not disclose the data collection dates, so it is unclear whether data were collected before 2002.

Williams et al. (2003) did not define adherence, as they assumed that physician participants could accurately report their adherence to current screening guidelines. Ross et al. (2008) categorized adherence according to risk category: women with higher risk of cervical cancer (sexually active women who have never received a Pap smear and women with a past abnormal Pap smear) should have had 1 Pap smear in the year prior to study enrollment, and women with lower risk (all women not meeting high-risk criteria) should have had 1 Pap smear in the 3 years prior to study enrollment.

**HIV-Positive Women**

The two studies that assessed screening participation in HIV-positive women evaluated different variables (Logan, Khambaty, D’Souza, & Menezes, 2010; Tello et al., 2010). Like the studies in the other population categories, the study by Logan et al. (2010) indicated that having health insurance was positively associated with screening adherence. Logan et al. (2010) also noted that the insurance type was associated with screening adherence, but the types were not reported.

Like the studies in the other populations, the study by Tello et al. (2010) revealed that older age and higher education were positively associated with cervical cancer screening adherence. Logan et al. (2010) used data collected before 2002. Logan et al. (2010) defined adherence to cervical cancer screening according to USPSTF recommendations for HIV-positive women. Women were considered adherent if they received two Pap smears in the first year after their HIV diagnosis, then annually. Tello et al. (2010) defined adherence as having received a Pap smear in the past year.

**Other Populations** 

Three studies included populations that were not classified similarly to any other studies in this review. The only finding that was common in two of these three studies is that age was associated with participation in screening (Camp et al., 2008; MacLaughlan, Lachance, & Gjelsvik, 2011; Tracy, Lydecker, & Ireland, 2010). MacLaughlan et al. (2011) concluded that women 21 to 64 years old were most adherent to screening guidelines and those 18 to 20 years old and 65 years and older were less adherent. In contrast, Tracy et al. (2010) found that older age was positively associated with screening adherence.

The study by MacLaughlan et al. (2011) had a large sample of 150,786 smokers, and random-digit dialing was used for participant selection. Adherence was defined as having had 1 Pap smear within the 3 years preceding study enrollment. The study by Tracy et al. (2010) had a relatively small sample of 225 lesbians, and the authors used nonrandom selection of participants. Adherence was defined as having had a Pap smear within the 2 years preceding study enrollment.

Camp et al. (2008) included a sample of 3,966 women who had been exposed to DES and were selected using convenience sampling. This unique study showed that DES-exposed women underwent more screening than non—DES-exposed women. Adherence was defined, per DES-specific guidelines, as having had at least 4 Pap smears in the previous 5 years. Of note, Camp et al. (2008) used data collected from 1990 to 1994.

## Commonalities Across Populations

Several variables were found to be associated with adherence to screening guidelines across population groups. Higher education level and financial status were significantly and positively associated with adherence rates in the majority of the studies reviewed. The consensus was that women with health insurance and higher income were significantly more likely to receive cervical screening. Seven studies concluded that young women were more adherent to screening guidelines (Bazargan et al., 2004; Borrayo et al., 2004; Carruth et al., 2006; Cook et al., 2010; Datta et al., 2005; Insinga et al., 2004; Tello et al., 2010), but four studies reported that older women were more adherent (Fernandez-Esquer et al., 2003; Lin et al., 2009; Ma et al., 2009; Tracy et al., 2010). These inconsistencies in age results may have arisen from variations in ages of the study populations and in grouping participants into younger and older categories.

Seven studies indicated that acculturation or duration of US residency was positively associated with participation in screening (Bazargan et al., 2004; Coronado et al., 2004; Fernandez-Esquer et al., 2003; Lin et al., 2009; Luque et al., 2011; Ma et al., 2009; Tsui et al., 2007), and seven studies showed that psychosocial issues such as major life events, mood disorders, and fear associated with screening were associated with participation in screening (Bazargan et al., 2004; Coronado et al., 2004; Ma et al., 2009; Nelson et al., 2009; Paskett et al., 2010; Tello et al., 2010; Tracy et al., 2010). Being married was positively associated with adherence to screening in seven studies as well, specifically in African Americans, Hispanic Americans, Caucasians, and Asian Americans (Carruth et al., 2006; Datta et al., 2005; Fernandez-Esquer et al., 2003; Lin et al., 2009; Luque et al., 2011; Ma et al., 2009; Taylor et al., 2004).

Four studies found that issues related to time, such as lack of time and scheduling problems, were associated with screening adherence (Coronado et al., 2004; Hoyo et al., 2005; Ma et al., 2009; Ross et al., 2008). Three studies found that smokers were significantly less likely to undergo screening than nonsmokers (Datta et al., 2005; MacLaughlan et al., 2011; Nelson et al., 2009), and two studies found that maintaining a healthy body weight (i.e., not being under- or overweight) was positively associated with screening adherence (Datta et al., 2005; Nelson et al., 2009). Two studies found that pain was a negative predictor of adherence to screening guidelines (Hoyo et al., 2005; Taylor et al., 2004). Two studies found that women who were adherent to breast cancer screening guidelines (mammography) were significantly more adherent to guidelines for cervical cancer screening than women not adherent to mammography guidelines (Borrayo et al., 2004; Carruth et al., 2006). Two studies showed that consistency in health care visits and provider recommendations for Pap smears were associated with participation in screening (Bazargan et al., 2004; Nelson et al., 2009).

## Discussion

Many variables emerged as predictors for use of cervical cancer screening. The most consistent findings were that a secure financial status or insurance, a higher level of education, marriage, a high level of acculturation, and good psychosocial health were positively associated with adherence to cervical cancer screening guidelines in US adult women.

Most of the studies that are included in this review were cross-sectional studies, which, by design, can reflect a correlation among variables but cannot determine causation. Several of the studies included data from only one clinic, hospital, or organization. The accuracy of those data could have been affected by the facility and its location. In the studies in which records were used, adherence may not have been accurately reflected.

Another potential problem with many of the studies was the reliance on self-report. Women may purposely or inadvertently over- or underreport screening consistency. In addition, results yielded in subgroup populations may not apply to the general population, even though the grouping of similar subjects does allow for control of some variables, such as place of residence, income, insurance status, or education level.

In several studies reviewed, data were collected before the 2002 ACS cervical cancer screening guidelines were published. Although all the studies were published after 2002, adherence calculations could have reflected minor differences in guidelines that were current at the time of data collection. Additionally, not all studies incorporated the same definition or timeframe for adherence. This discrepancy is reasonable, because women in different age groups and risk categories had different screening requirements, but it complicates comparisons of adherence among populations. Likewise, the studies did not measure the same independent variables, thereby making it difficult to determine whether some variables would have been more or less significant had they been studied in all populations.

This review ultimately elucidates several variables associated with adherence to cervical cancer screening, including educational level, financial status, acculturation, psychosocial issues, and marital status, among others (see Table). With this knowledge, nurse practitioners and other health-care providers can target populations for focused interventions to increase adherence to the 2012 screening recommendations. For example, one may develop an educational intervention for uninsured women who do not have a college degree. Also, nurses and other health-care providers may place special emphasis on screening importance and guidelines in populations such as Asian American women and others who may be less likely to undergo screening.

The Pap smear is a quick, easy, and relatively inexpensive test, and as such, its importance for health maintenance should be taught and understood by women in all socioeconomic populations. Advanced practitioners and all primary health-care providers should understand and make efforts to minimize barriers to screening. These efforts may include, for example, promoting opportunities for free screening to women with cost barriers and establishing opportunities for late or weekend screening for those with time constraints. Regardless of the limiting barrier that might be present, health-care providers are responsible for educating the public about the necessity and usefulness of the Pap smear and for ensuring that women at risk for cervical cancer are afforded screening opportunities to enable early diagnosis, successful treatment, and even prevention of this disease. l
